# Sleep Quality among Female Hospital Staff Nurses

**DOI:** 10.1155/2013/283490

**Published:** 2013-05-13

**Authors:** Pei-Li Chien, Hui-Fang Su, Pi-Ching Hsieh, Ruo-Yan Siao, Pei-Ying Ling, Hei-Jen Jou

**Affiliations:** ^1^Department of Preventive Medicine, Taiwan Adventist Hospital, No. 424, Section 2, Bade Road, Songshan District, Taipei 105, Taiwan; ^2^Department of Health Care Management, National Taipei University of Nursing and Health Sciences, No. 89, Nei-Chiang Street, Wanhua District, Taipei 10845, Taiwan; ^3^Department of Obstetrics and Gynecology, Taiwan Adventist Hospital, No. 424, Section 2, Bade Road, Songshan District, Taipei 105, Taiwan; ^4^Department of Obstetrics and Gynecology, National Taiwan University Hospital, No. 7, Zhongshan S. Road, Zhongzheng District, Taipei 100, Taiwan

## Abstract

*Purpose*. To investigate sleep quality of hospital staff nurses, both by subjective questionnaire and objective measures. *Methods*. Female staff nurses at a regional teaching hospital in Northern Taiwan were recruited. The Chinese version of the pittsburgh sleep quality index (C-PSQI) was used to assess subjective sleep quality, and an electrocardiogram-based cardiopulmonary coupling (CPC) technique was used to analyze objective sleep stability. Work stress was assessed using questionnaire on medical worker's stress. *Results*. A total of 156 staff nurses completed the study. Among the staff nurses, 75.8% (117) had a PSQI score of ≥5 and 39.8% had an inadequate stable sleep ratio on subjective measures. Nurses with a high school or lower educational degree had a much higher risk of sleep disturbance when compared to nurses with a college or higher level degree. *Conclusions*. Both subjective and objective measures demonstrated that poor sleep quality is a common health problem among hospital staff nurses. More studies are warranted on this important issue to discover possible factors and therefore to develop a systemic strategy to cope with the problem.

## 1. Introduction

Poor sleep quality among hospital stuff nurses is a critical issue for healthcare system. It not only leads to health problems of the nurses, but it is also associated with a lower work performance and a higher risk of medical errors which may jeopardize patient's safety [1]. The incidence of sleep disturbance among general Asian population ranged from 26.4% to 39.4% [2, 3]. Most previous studies on sleep quality of nurses focused on the effect of shift work on subjective sleep perception using self-report questionnaire and revealed that up to 57% of shift-working nurses had sleep disturbance [4]. It is therefore warranted to find out possible factors associated with sleep disturbance of working nurses. 

The perception of sleep quality is complex and associated with various subjective factors such as fatigue, work stress, or other emotional factors in addition to objective sleep quality. However, limited study of objective sleep quality of hospital nurses provides little information for better understanding of sleep disturbance among this population. 

The current methods used to assess objective sleep physiology primarily rely on polysomnography (PSG) which is based on the analysis of signals of electroencephalography (EEG), electrooculography, electromyography, and electrocardiography (ECG). Although PSG is the gold standard for objective sleep quality assessment, the cost and technical complexity of PSG limit its use as a routine screening tool. In addition, it has been noted that PSG measurement may have been largely affected by “first-night effect” from an unfamiliar laboratory environment and discomfort from PSG sensors and equipment [5]. In addition, sleep pattern of subjects at home may be quite different when compared with that in the laboratory room. Previous studies have also demonstrated inconsistency between subjective sleep perception and PSG staging [6–8]. 

Recently, an alternative method term cardiopulmonary coupling (CPC) analysis has been developed to quantify sleep stability. The technique is based on analysis of continuous ECG signals using take-home ECG devices [9]. This novel technology is less expensive and is more convenient since the measures do not require subjects to sleep in a laboratory room. Therefore the study does not interrupt the daily work of the nurses. 

The purpose of this study was to assess the sleep quality of hospital stuff nurses, including subjective sleep perception by self-report questionnaire and objective sleep stability by CPC method. We also tried to identify possible demographic, lifestyle, and work factors for poor sleep quality among hospital working nurses.

## 2. Methods

### 2.1. Subjects

A cross-sectional study was conducted from March 1, 2009 to September 30, 2009 to investigate the subjective sleep perception, objective sleep stability, and the possible factors associated with poor sleep quality among staff nurses at a regional teaching hospital with 389 nurses. One hundred and seventy-five subjects from the hospital, including nurses and nurse managers, were randomly selected by computer chaos generator with an approximate nurse/nurse manager ratio of 3 : 1 and study subjects comprising about half of the total hospital nursing staff and nursing managers. Male nurses were excluded from the study because of their small proportion among the nursing staff. Written informed consent from each subject was obtained after the study's approval by the Institutional Review Board of the study hospital. 

 The authors developed a detailed questionnaire according to the literature, which included demographic information (age, gender, education level, marital status, etc.) and associated factors such as regular exercise, managerial position, and work shift. Regular exercise was defined as a regular exercise habit of ≥3 exercise per week with duration of ≥30 minutes every time. Work shift was defined as >1 work shift per week during last month. 

### 2.2. Assessment of Work Stress

Questionnaire on medical worker's stress (QMWS) (Cronbach alpha coefficient 0.84) was used to assess the subjects' work stress [10]. There are eight questions in the QMWS, including stress from (1) running the hospital, (2) preparing the accreditation of the hospital, (3) the stability of the patients' condition, (4) the relationship with patients, (5) medical dispute or lawsuits, (6) salary, (7) personal assessment, and (8) position upgrade or academic research. Each item scored from 1 (very sure not to be a stress factor) to 6 (very sure to be a stress factor) with a total score ranging from 8 to 48. The subjects were subdivided into two groups: the higher stress group (HSG) with a score of >32 and the lower stress group (LSG) with a stress score of ≤32. 

### 2.3. Assessment of Subjective Sleep Quality

The Chinese edition Pittsburgh sleep quality index (C-PSQI) was used to assess subjective sleep quality [11]. Subjects were asked to assess their sleep condition in the previous month. The PSQI is a self-rated questionnaire and comprises 18 questions to assess seven domains of sleep, including subjective sleep quality, sleep latency, sleep duration, habitual sleep efficiency, sleep disturbances, use of sleeping medication, and daytime dysfunction. Each domain is rated on a 4-point scale (0–3), which generates a total score ranging between 0 and 21. Poor sleepers had a total score of 5 or above, while good sleepers had a score of less than 5. The Cronbach's *α* values were 0.83 for the original PSQI instrument [12] and 0.77 for C-PSQI [11], respectively. 

### 2.4. Assessment of Objective Sleep Quality

A take-home Holter ECG SD-100 portable recorder (Microstar Inc., Taipei, Taiwan) was used to obtain continuous ECG signals of the subjects during their time in bed with only 4 leads attached to the trunk of the subjects. All subjects received ECG recording at home after a detailed demonstration of the recording process by the researcher. The ECG recording was collected on the second day and then uploaded to a central laboratory. The signals were automatically processed and analysed to generate a sleep pictogram using an ECG-based CPC (cardiopulmonary coupling analysis) technique [9]. Three physiological sleep states were classified based on CPC analysis: stable sleep, unstable sleep, and REM/wakeful (awake/dream) states [9], as expressed by both absolute time duration and ratio of time in bed. A stable sleep ratio ≥41% was regarded as adequate sleep “stability”, while inadequate sleep stability indicated a stable sleep ratio of <41%. Recorded data also included total sleep time, sleep latency duration, and apnea/hypopnea index (AHI). 

In addition to the three physiological sleep patterns, the machine also records sleep apnea/hypopnea of the subjects. The AHI represents an index of apnea/hypopnea per hour of time in bed. An AHI of 5 to 15 was defined as mild apnea/hypopnea, an AHI between 16 and 30 meant moderate apnea/hypopnea, and an AHI of greater than 30 signified severe apnea/hypopnea.

### 2.5. Statistic Analysis

Descriptive analysis was used to analyze basic characteristics of the subjects with mean values ± standard deviation (*SD*). Comparison between groups was done with Student's *t*-test for continuous variables and *χ* ^2^ test for discrete variables. Multiple variable logistic regression was used to calculate the odds ratios of variant demographic factors on subjective perception and objective sleep stability. Linear regression was used to identify the association between PSQI and parameters of objective measures. We considered the differences were significant at *P*< 0.05 for all statistical tests. All analyses were performed with the SPSS 17.0. (Allyn & Bacon, Inc., Needham Heights, MA, USA). 

## 3. Results

### 3.1. Subjects

During the study period, 19 subjects dropped out, including 6 of 44 nurse managers and 13 of 131 first-line staff nurses, which resulted in a response rate of 89.1% (156/175). The reasons for the dropout included, worrying about sleep interruption by the equipment (10), unwillingness to do the test (6), and personal issues (3). There were two subjects who failed their initial CPC recording, and a second test was performed.

### 3.2. Demographic Data and Sleep Assessment

The subjects' mean age and years of nursing work experience were 34.6 ± 8.1 and 7.9 ± 7.2 years, respectively. Half of the subjects (50.0%) were single. Nurse managers had a higher percentage of college or higher level of education when compared with that of nonmanager nurses (68% versus 37%, *P* = 0.01).  Table 1 summarized basic characteristics of the subjects. Forty-five percent of the nurses had less five years of nursing experience, and 50.6% had a QMWS score of >32.  Table 2 demonstrated subjective and objective sleep measures of the subjects. Mean PSQI score was 7.34 ± 2.94. Objective measures showed a mean stable sleep ratio of 46.62 ± 17.26% and a mean AHI of 4.32 ± 7.68.

A comparison among variable factors with sleep measures by logistic regression is summarized in Table 3. Sleep assessments revealed that 75.0% (117/156) of subjects had a total PSQI score of 5 or above, which indicated a significant sleep problem, and 39.7% of subjects (62/156) had inadequate sleep stability according to CPC records. Comparison between the basic characteristics and the sleep measures showed that years of nursing work experience, age, marital status, number of child, habit of regular exercise, work position, work shift, and work stress were not significantly related to a poor subjective sleep perception or an inadequate stable sleep ratio. However, there was significant relationship between the education levels and both subjective sleep perception and objective sleep stability. Nurses with educational level of high school or under had odds ratios of 2.69 to be a poor sleeper (*P* = 0.02) and of 2.71 to have an inadequate sleep stability (*P* = 0.01). 

Linear correlation analysis revealed that PSQI score was weakly but significantly correlated with the unstable sleep duration (*r* = 0.20, *P* = 0.01), unstable sleep ratio (*r* = 0.22, *P* = 0.006), and AHI frequency (*r* = 0.25, *P* = 0.002) (Table 4). A significant reverse correlation between stable sleep ratio and PSQI score (*r* = 0.17, *P* = 0.03) was observed as well. There were no significant correlations between PSQI score and awake/dream duration (*r* = 0.04, *P* = 0.58), awake/dream ratio (*r* = 0.02, *P* = 0.89), and sleep latency duration (*r* = 0.04, *P* = 0.59). 

## 4. Discussion

The present study has demonstrated that nurses working in a hospital setting had a high prevalence of sleep disturbance. Most previous studies were based on subjective questionnaire. However, our data provided objective evidence, which is less affected by chronic fatigue or subjective perception on this important issue. In addition, the present study revealed that nurses with educational level of high school or under had a much higher risk of sleep disturbance when compared with nurses with a college or higher degree. There were no significant associations between sleep disturbance and years of nursing experience, age, marital status, number of children, regular exercise habit, manager position, work shift, or work stress.

The present study revealed that neither subjective nor objective sleep quality was related to shift work. Although some previous studies have revealed that shift work schedule contributed one of the major causes of subjectively poor sleep quality of nurses [4, 13], the conclusions were inconsistent. The study by Sveinsdóttir failed to show such relationship using a questionnaire called “Women's Health” [14]. It has been proposed that adequate shift assignment and stress reduction can prevent pathologically disrupt circadian cycle and sleep disturbance [14], but further studies are needed to clarify this hypothesis. 

Only limited number of studies has examined the relationship between sleep disturbance and educational level of nursing staff. The present study demonstrated that nurses with an educational level of high school or under had two times higher incidence of poor sleep quality when compared with nurses with an educational level of college or higher degree. These results were compatible with previous studies [15, 16], in which the authors proposed that nurses with higher educational level had a less work-related stress [15] and less emotional exhaustion [16]. But the present study failed to show the association between work stress and poor sleep quality. 

Hospital staff nurses with moderate/severe sleep apnea/hypopnea did not have significantly higher incidence of poor sleep perception when compared with nurses with less AHI. To our best knowledge, no previous study investigated the incidence of sleep apnea/hypopnea among hospital staff nurses. The results of the present study were compatible with previous studies on subjects with severe obstructive sleep apnea (OSA) [17–19]. Possible explanations for failure of association between the degree of AHI and subjective sleep perception might include the factors other than OSA affect subjective sleep quality. Additionally, AHI scoring was based on the frequency of apnea/hypopnea instead of severity of OSA, but poor sleep perception was more related to the frequency of arousal caused by OSA. However, only nine nurses had an AHI > 15 in the present study. Study on a larger population is needed to get further conclusion on this issue.

Objective measurement may be affected by instruments, resulting in differences of sleep condition between test days and ordinary days. This was the reason for which this study opted to use home instruments. However, although the prevalence of the “first-night effect” caused by environmental changes may be prevented, sleep may be disturbed by electrodes being stuck to their bodies. The present study demonstrated that the ECG-based measures were likely to prevent the first-night effect because only two subjects reported that their sleep was bothered by the devices and needed a second measurement. Further studies comparing the positive and the negative predictive value of the CPC against the PSG measures may be helpful on this issue.

The present study revealed that the relationship was weak, though being significant, between PSQI score and variant objective parameters by CPC measures. The results indicate that the two measures may get different results, and that they cannot be substituted for each other. This study used PSQI questionnaires to investigate the sleep conditions of subjects over the previous month, and therefore special events or differing sleep perceptions may influence the test results. The objective measurement conducted by this study can only present the sleep conditions on the day of testing. Although the results showed decreased stable sleep ratio, increased unstable sleep duration, increased unstable ratio, and higher AHI frequency may be indicators for a poor subjective sleep quality, gaps are found in terms of objective and subjective sleep evaluations. Nurses had higher incidence of poor PSQI than of inadequate objective sleep stability. Factors other than poor objective sleep stability may also contribute to a poor PSGI score, which can explain the discrepancy between objective and subjective measures.

Several limitations of this study need to be taken into account when making conclusions. First of all, there is no data from a nonmedical general population as a control group in the study design to compare the difference of sleep disturbance between hospital staff nurses and the general population. Secondly, survey from one hospital would limit the applicability and generalization of study findings to other hospitals with different level. Furthermore, the study did not clarify the type of work shift and did not include possible factors such as chronic fatigue or other emotional factors. It is very difficult to assess all possible factors in one single study. Further studies are needed on this issue. However, the present study did reveal that it can be very helpful when used as an objective measure as an adjuvant of self-report questionnaire to assess sleep quality in spite of the difference in the nature of the two tests. 

In conclusion, the present study showed that poor subjective sleep quality and poor objective sleep stability both occur frequently among hospital nursing staff. Some objective measures that are easily applicable can be used to assist the interpretation of subjective data. Educational level of high school or lower degree is the only predictor for poor sleep quality of the nurses in the present study. This finding provides important information for hospitals in Taiwan. Poor sleep quality remains a vital health issue for hospital staff nurses, and in-service education may be helpful for reducing sleep disturbance in this population. However, further research on this subject using larger populations is needed to reach an appropriate conclusion. 

## Figures and Tables

**Table 1:** Basic characteristics of the subjects.

Variable	*N *	%
Total	156	100
Nursing experience		
<5 years	70	44.9
≥5 year	86	55.1
Age		
<40 years	115	73.7
≥40 years	41	26.3
Education level		
College or higher degree	70	44.9
High school or lower degree	86	55.1
Marital status		
Married	78	50.0
Single	78	50.0
Number of children		
≥1	63	40.4
None	93	59.6
Regular exercise		
Yes	77	47.8
No	79	50.6
Nurse manager		
Yes	38	24.4
No	118	75.6
Work shift		
Yes	49	32.3
No	107	67.7
Working pressure		
Lower (8–32)	79	50.6
Higher (33–48)	77	49.4

Note.   * Work pressure: measured by questionnaire of medical worker's stress (QMWS): lower work pressure: score 8–32; higher work pressure: 33–48.

**Table 2:** Subjective and objective sleep measures of the subjects.

Sleep indices	Mean	SD
PSQI (score)	7.34	2.94
Subject sleep quality (score)	1.25	0.77
Sleep latency (minute)	18.33	13.09
Sleep duration (hours)	6.11	1.22
Sleep efficiency (%)	83.20	13.81
Sleep disturbance (score)	6.54	3.41
Use of sleep medications (score)	0.23	0.63
Daytime dysfunction (score)	1.91	1.43
CPC		
Sleep latency (minute)	26.79	43.80
Stable sleep ratio (%)	46.62	17.26
Unstable sleep ratio (%)	29.78	13.34
Awake/dream sleep (%)	22.26	8.01
AHI	4.32	7.68

Note.  PSQI: Pittsburgh sleep quality index; CPC: cardiopulmonary coupling analysis, AHI: apnea/hypopnea index.

**Table 3:** The relationship between variable factors in subjective or objective poor sleep quality.

Variable	PSQI*				CPC**			
	Poor sleeper	Good sleeper	OR	*P*	Inadequate sleep stability	Adequate sleep stability	OR	*P*
*N* (total = 156)	117	39			62	94		
Working years								
<5 years	56	14			26	44		
≥5 year	61	25	0.40	0.06	36	50	0.81	0.63
Age								
<40 years	85	30			41	74		
≥40 years	32	9	1.64	0.34	21	20	0.91	0.85
Education level								
College or higher degree	47	23			34	36		
High school or lower degree	70	16	2.69	0.02	28	58	2.71	0.01
Marital status								
Married	58	20			32	46		
Single	59	19	0.67	0.60	30	48	0.78	0.71
Number of Children								
≥1	46	17			26	37		
None	71	22	0.60	0.49	36	57	0.98	0.97
Regular exercise								
Yes	56	21			27	50		
NO	61	18	1.08	0.85	35	44	0.72	0.38
Nurse manager								
Yes	28	10			14	24		
No	89	29	0.67	0.43	48	70	0.53	0.17
Work shift								
Yes	36	13			17	32		
No	81	26	0.90	0.82	45	62	1.90	0.16
AHI								
≦15	109	38			53	94		
>15	8	1	2.34	0.45	9	0	NA	NA
Working pressure^#^								
Lower (8–32)	56	21			25	52		
Higher (33–48)	61	18	1.68	0.21	37	42	0.49	0.07

*Note. *PSQI: pittsburgh sleep quality index, good sleeper had a PSQI <5 and a poor sleeper had a PSQI ≥5; CPC: cardiopulmonary coupling analysis, a stable sleep ratio ≥41% indicates adequate sleep stability and a stable sleep ratio <41% indicates inadequate sleep stability; ^#^Work pressure measured by questionnaire of medical worker's stress (QMWS). Multiple variable logistic regression was used for statistical analysis.

**Table 4:** Correlation between objective sleep indices and PSQI score.

	PSQI score		
	*β*	*r*	*P*
Sleep latency duration (minute)	0.003	0.04	0.59
Stable Sleep ratio (%)	−0.03	0.17	0.03
Unstable Sleep duration	1.26	0.20	0.01
Unstable Sleep ratio (%)	0.05	0.22	0.006
Awake/Dream duration	0.26	0.04	0.58
Awake/Dream ratio (%)	−0.004	0.01	0.89
AHI frequency	0.09	0.25	0.002

PSQI: pittsburgh sleep quality index; CPC: cardiopulmonary coupling analysis; AHI: Apnea-hyponea Index.

## References

[1] Gaba DM, Howard SK (2002). Fatigue among clinicians and the safety of patients. *The New England Journal of Medicine*.

[2] Doi Y, Minowa M, Uchiyama M, Okawa M (2001). Subjective sleep quality and sleep problems in the general Japanese adult population. *Psychiatry and Clinical Neurosciences*.

[3] Wong WS, Fielding R (2011). Prevalence of insomnia among Chinese adults in Hong Kong: a population-based study. *Journal of Sleep Research*.

[4] Shao MF, Chou YC, Yeh MY, Tzeng WC (2010). Sleep quality and quality of life in female shift-working nurses. *Journal of Advanced Nursing*.

[5] Mendels J, Hawkins DR (1967). Sleep laboratory adaptation in normal subjects and depressed patients (“first night effect”). *Electroencephalography and Clinical Neurophysiology*.

[6] Conroy DA, Todd Arnedt J, Brower KJ (2006). Perception of sleep in recovering alcohol-dependent patients with insomnia: relationship with future drinking. *Alcoholism*.

[7] Feige B, Al-Shajlawi A, Nissen C (2008). Does REM sleep contribute to subjective wake time in primary insomnia? A comparison of polysomnographic and subjective sleep in 100 patients. *Journal of Sleep Research*.

[8] Kushida CA, Chang A, Gadkary C, Guilleminault C, Carrillo O, Dement WC (2001). Comparison of actigraphic, polysomnographic, and subjective assessment of sleep parameters in sleep-disordered patients. *Sleep Medicine*.

[9] Thomas RJ, Mietus JE, Peng CK, Goldberger AL (2005). An electrocardiogram-based technique to assess cardiopulmonary coupling during sleep. *Sleep*.

[10] See LC, Chang HJ, Liu MJ, Cheng HK (2007). Development and evaluation of validity and reliability of a questionnaire on medical workers’ stress. *Taiwan Journal of Public Health*.

[11] Tsai PS, Wang SY, Wang MY (2005). Psychometric evaluation of the Chinese version of the Pittsburgh Sleep Quality Index (CPSQI) in primary insomnia and control subjects. *Quality of Life Research*.

[12] Buysse DJ, Reynolds CF, Monk TH, Berman SR, Kupfer DJ (1989). The Pittsburgh Sleep Quality Index: a new instrument for psychiatric practice and research. *Psychiatry Research*.

[13] Rutledge T, Stucky E, Dollarhide A (2009). A real-time assessment of work stress in physicians and nurses. *Journal of Health Psychology*.

[14] Sveinsdóttir H (2006). Self-assessed quality of sleep, occupational health, working environment, illness experience and job satisfaction of female nurses working different combination of shifts. *Scandinavian Journal of Caring Sciences*.

[15] Lu H, While AE, Louise Barriball K (2007). A model of job satisfaction of nurses: a reflection of nurses’ working lives in Mainland China. *Journal of Advanced Nursing*.

[16] Alimoglu MK, Donmez L (2005). Daylight exposure and the other predictors of burnout among nurses in a University Hospital. *International Journal of Nursing Studies*.

[17] Kezirian EJ, Harrison SL, Ancoli-Israel S (2009). Behavioral correlates of sleep-disordered breathing in older men. *Sleep*.

[18] Macey PM, Woo MA, Kumar R, Cross RL, Harper RM (2010). Relationship between obstructive sleep apnea severity and sleep, depression and anxiety symptoms in newly-diagnosed patients. *PLoS ONE*.

[19] Naismith S, Winter V, Gotsopoulos H, Hickie I, Cistulli P (2004). Neurobehavioral functioning in obstructive sleep apnea: differential effects of sleep quality, hypoxemia and subjective sleepiness. *Journal of Clinical and Experimental Neuropsychology*.

